# Patient autonomy, clinical decision making, and the Phenomenological reduction

**DOI:** 10.1007/s11019-022-10102-2

**Published:** 2022-07-07

**Authors:** Jonathan Lewis, Søren Holm

**Affiliations:** 1 The Centre for Social Ethics and Policy, Department of Law, School of Social Sciences, The University of Manchester, Oxford Road, M13 9PL Manchester, UK; 2 Centre for Medical Ethics, Faculty of Medicine, University of Oslo, Oslo, Norway

**Keywords:** Phenomenology, Patient autonomy, Relational autonomy, Clinical decisions, Authenticity

## Abstract

Phenomenology gives rise to certain ontological considerations that have far-reaching implications for standard conceptions of patient autonomy in medical ethics, and, as a result, the obligations of and to patients in clinical decision-making contexts. One such consideration is the *phenomenological reduction* in classical phenomenology, a core feature of which is the characterisation of our primary experiences as immediately and inherently meaningful. This paper builds on and extends the analyses of the phenomenological reduction in the works of Husserl, Heidegger, and Merleau-Ponty in order to identify and explain its implications for our current understanding of the principle of respect for patient autonomy and the norms of clinical decision making.

For patients, being recognised as autonomous in the context of clinical decision making has normative significance for two reasons. Firstly, it sets the parameters within which they should be immune from paternalistic clinical interventions. In both medical law and biomedical ethics, informed consent is the standard mechanism through which a patient establishes the boundaries of her sovereignty (Archard 2008; Walker 2013; Lewis 2020). However, the right to partake in practices of informed consent (without third party involvement) is only extended to adult patients who are presumed to fulfil the demands of mental capacity. As Wayne Martin and Ryan Hickerson (2013) observe, capacity assessments in recent years have affirmed a particular approach to autonomy based on the satisfaction of conditions of *competency* linked to individual cognitive performance. For example, the test for incapacity in Sect. 3(1) of the Mental Capacity Act 2005 in England and Wales raises a strong, albeit negative, affirmation of the capacity for autonomy, whereby an individual is unable to make a decision if they are unable to understand, retain, use, or weigh information relevant to a decision or communicate that decision. The implication is that when a patient lacks deliberative competence capacities and thereby is taken to lack the capacity for autonomy, outside interference in the decision-making process is more likely to be justified.

“Competency,” “mental capacity,” and the “capacity for autonomy” are often treated as having the same meanings such that, on the basis of a certain amount of consensus amongst medical ethicists, a competent or capacitous individual is considered to have the “capacity for reason,” that is, “the capacities to comprehend information, critically reflect on and revise beliefs, and make a decision in the light of information” (Lewis 2021a, p. 16). Ultimately, the competency approach to autonomy in both medical law and ethics operates on the basis that individual cognitive capacities for reason are necessary constituents of a patient’s *capacity* for autonomous decision making in clinically related contexts (Foddy and Savulescu 2006; Holroyd 2009; Schaefer et al. 2014; Lewis 2021a). When combined with liberal principles, the concept of the competent agent has been the backbone of regulatory and statutory approaches to medical decision making (Lewis 2021a, p. 16). However, the fact that an individual has been accorded the liberty to consent on the basis of her (presumed) competency/mental capacity does not guarantee that the consent she provides will be autonomous because there are no assurances that she has, as a matter of fact, understood or rationally deliberated on the information with which she has been informed or the knowledge she possesses. Thus, although satisfactory fulfilment of the conditions for competency (i.e., the capacity for reason) accounts for an individual’s autonomous *agency* (i.e., their *capacity for autonomy*), it does not ensure that an individual makes an autonomous *choice*.

There is, therefore, a principled distinction between a patient’s *capacity for autonomy* and their *exercise of autonomy* (Lewis 2021a, b), and this distinction will prove to be vital for understanding the implications of our approach to the phenomenological reduction for clinical decision making as well as the relationships between a more phenomenologically oriented approach to patient autonomy and traditional theories of autonomy, both of which are the focus of this paper. But before we broach this topic, it is important to acknowledge that this distinction points to a second reason why recognition of patient autonomy is normatively significant. Specifically, where the notion of autonomous choice or the exercise of autonomy is concerned, respect for autonomy allows competent patients to effect changes in their lives in a manner that is consistent with their values, desires, and motivations. As Coggon and Miola observe, when we are discussing whether an agent is exercising their autonomy, “there is a concern not just for the capacity for reason, but also for the effective use of it” (Coggon and Miola 2011, p. 528).^1^ In short, whether a patient’s exercise of their autonomy qualifies as “effective” will depend on “the soundness of her reasoning, given her own values” (ibid., p. 531) or, more broadly construed, the extent that she is the “power” behind whatever reasoning directly gives rise to her decisions, choices or actions (Buss and Westlund 2018). Although there are philosophical disagreements about what exactly constitutes “sound reasoning” and the nature of the “power” over such reasoning, the point is that an agent’s values, desires, and reasons “can be more or less autonomous depending on whether the processes or volitional structures by which they come to be developed are *truly* her own” (Lewis 2021a, p. 18). In terms of theories of autonomy, these processes and structures have traditionally been framed in purely cognitive terms, that is, as processes of introspective, critical reflection or as endorsements, identifications with, or rational responses to one’s values, desires, and motives by way of an appropriate cognitive mechanism (Christman 2004).

Conceptions of the autonomous patient as a cognitively capacitous, self-sufficient, and introspective individual, and associated theories of autonomy that appeal to rational reflection as the preeminent arbiter of autonomy, have been perceived as problematic, both conceptually and when applied in practice. Commentators have demonstrated that, when it comes to medical decisions, theories of autonomy that focus solely on the cognitive capacities for reason and conditions of rational reflection fail, in practice, to adequately capture the autonomy of certain individuals and groups despite individuals within those groups fulfilling the standards for mental capacity (Clough 2014, 2017; Herring and Wall 2015; Lewis 2021b). In addition, by discounting the impact of social and interpersonal relations on one’s exercises of autonomy (e.g., see Meyers 1989; Benson 1991; Mackenzie and Stoljar 2000; Meyers 2000; Friedman 2003; Christman 2004; 2009), appeals to traditional theories of autonomy preclude the possibility that other persons and social institutions may be obligated to help restore, support, or promote an individual’s autonomy (Mackenzie 2008; Dodds 2014; Mackenzie et al. 2014). As a result, theorists have argued for more *relational* accounts of autonomy (e.g., Meyers 2005; Mackenzie 2008; Westlund 2009; Anderson 2014; Mackenzie 2014; 2015; Westlund 2018), which are premised on an understanding of interpersonal and social relationships as—depending on the type of approach to relational autonomy—constitutive of *autonomous agency* or the background conditions that causally affect the *exercise and achievement of autonomy* (Mackenzie 2021). However, even though relational theorists are concerned with the ways in which interpersonal and social relations can support or undermine the conditions for autonomy, many of the most established accounts are still committed to a notion of autonomy understood purely as the exercise of certain cognitive capacities for reason (Meyers 2000, 2005). It follows that while relational theorists reject the valorisation of the integrated, self-sufficient, and introspective individual, they – to varying degrees – share the non-relationalist’s commitment to the autonomous agent who turns her own attitudes into objects of reflection to determine how she will *exercise* her autonomy.^2^

On the surface, phenomenology might be perceived to offer support for the sort of introspective reflection that constitutes an individual’s exercise of her capacity for autonomy. After all, as Gallagher (2012, p. 58) notes, a standard objection to phenomenology is that it is a form of introspection and thereby a subjective exercise. However, this objection misses the point of classical phenomenology, as espoused by Husserl, Heidegger, and Merleau-Ponty (among others).

In this paper, we begin by demonstrating that this “introspective” interpretation of the nature of phenomenology is challenged by the notion of the “phenomenological reduction,” according to which, by suspending the natural way we tend to judge and look at reality, we acknowledge that our pre-reflective experiences of and in the world are inherently meaningful. Furthermore, the upshot of the phenomenological reduction is that such meaningfulness is encountered initially through first-person embodied and affective intentional experiences rather than through cognitive acts. As we will demonstrate, this calls into question the ability of non-relational and relational theories of autonomy, and their application in medical ethics and medical law, to fully capture patient autonomy, focused as they are on the satisfaction of cognitive conditions for mental capacity and the exercise of those capacities in acts of introspective, rational reflection, identification, or endorsement.

In section two, we argue that the phenomenological reduction, and a phenomenologically oriented approach to autonomy that it entails, cannot be entirely squared with traditional theories of autonomy. Moreover, and as we discuss in greater detail in section three, we also demonstrate that an approach to patient autonomy based on the phenomenological reduction is not compatible with exclusively relational accounts of autonomy. Therefore, one of novelties of this paper lies in the fact that it not only calls into question the practical adequacy of traditional theories of autonomy, and their application in medical ethics and medical law, but also that it does so without necessarily relying on a relational conception of autonomy. In addition, the analysis in section two challenges standard approaches to patient autonomy in clinical decision-making contexts. Our contribution to the literature on patient autonomy is based on an understanding of the ways in which the phenomenological reduction discloses how one makes sense of one’s autonomy-determining values, desires, and motivations at a pre-reflective level, particularly through moods, emotions, feelings, and active bodily engagements within our environments. We argue that not only are one’s capacities for affective and bodily intentional experience necessary components of one’s capacity for autonomy, but also one’s exercises of autonomy when making treatment decisions and choices are necessarily dependent upon, and can be constituted by, manifestations of one’s capacities for bodily and affective intentionality in certain affective states and skilful practices. Thus, we argue that not only is the phenomenological reduction a condition for the experience of autonomy and experience in general, but also the affective and embodied dimensions of experience can be integral parts of a patient’s exercise of their autonomy. Throughout sections two and three, we explain the key practical implications of the phenomenological reduction and affective and bodily intentional experience for clinical approaches to patient autonomy in medical decision making.

## Phenomenological Reduction: Towards a Functional Definition

Before we explore the implications of the phenomenological reduction for clinical decision making and the relationships between a more phenomenologically oriented conception of autonomy and traditional theories of autonomy, we need to understand what the phenomenological reduction is and how it intervenes on questions of intentionality, experience, and, relatedly, the distinctions between rationality and irrationality, reason and emotion, and mind and body. This task is complicated by the fact that, when it comes to individual classical phenomenologists, not only are there conflicting interpretations of the phenomenological reduction, but also the respective approaches to the phenomenological reduction in, on the one hand, the works of Husserl and, on the other, those of Heidegger and Merleau-Ponty have traditionally been viewed as incompatible (Smith 2005). Since accounting for the nuances of and between these accounts and interpretations is beyond the scope of this paper, we attempt, in this section, to identify some common characteristics of the phenomenological reduction supported by exegetical work in the secondary literature.^3^

As we shall explain in the next section, theories of autonomy that appeal solely to individual cognitive capacities, and the exercise of these capacities in rational reflection, are problematic not least because they preclude consideration of the affective and embodied dimensions of experience. The primary aim of this paper is to explain the ways in which non-cognitive capacities for experience, and the exercise of these capacities when they manifest as moods, feelings, emotions, and active bodily engagements, can be integral to a patient’s autonomy. On that basis, our characterisation of the phenomenological reduction relies more on the approaches adopted by Heidegger and Merleau-Ponty, for whom affectivity and embodied practical engagements are, in varying degrees, constitutive of phenomenologically reductive acts of sense-making, rather than those of Husserl or other classical phenomenologists.

It wouldn’t be appropriate to explicate this approach without briefly considering a core component of Husserl’s phenomenological reduction, to which both Heidegger and Merleau-Ponty responded. For Husserl, the phenomenological reduction is a technique, constituted by two devices, that provides us with access to the world of *phenomena*, as well as specific *phenomena*, in contrast to objective, mind-independent reality, which has been assumed to be the focus of scientific enquiry and epistemic claims made in the natural sciences. Broadly, there is a distinction to be made between the phenomenological way of looking at the world and the “natural” way (Husserl, 1982 § 32). Achievement of the former requires, firstly, a device by which we suspend or bracket the natural way in which we judge and look at reality (*epoché*), and, secondly, the reduction proper by which we (phenomenologically) inquire into an aspect of suspended reality (Smith 2005, p. 555). According to Husserl, once we have bracketed the intentional content that comes with our “natural” way of judging and looking at reality, what is left is the “phenomenological residuum” of “absolute consciousness” (i.e., those aspects of our intentional acts and their contents that do not depend on the existence of a represented object “out there” in mind-independent reality) (Husserl, 1982 § 50).

Although it is common to claim that both Heidegger and Merleau-Ponty reject the phenomenological reduction,^4^ more recently others have argued that they maintain the two devices employed by Husserl, namely, the *epoché* and the reduction proper (Smith 2005; Sheehan 2014). However, whereas Husserl’s conception of the phenomenological reduction entails phenomenological inquiries into intentional contents at the level of “absolute consciousness” (a matter that we do not need to take up here), Heidegger and Merleau-Ponty’s respective conceptions involve an understanding of intentionality, that is, the *directedness* or *aboutness* of our corporeal movements, perceptions, judgments, feelings, and thoughts, at the level of “being-in-the-world”.^5^ Broadly speaking, we are meaningfully in the world and the world is meaningfully in us even before we turn it into an object of reflection.

According to Thomas Sheehan, based on Heidegger’s interpretation of intentionality in terms of “being,” “things out there in the universe come to be seen as meaningfully present phenomena: the perceived of a perception, the loved of an act of love, the judged of an act of judgment—that is, always in correlation with a human concern or practice” (Sheehan 2014, p. 128). A phenomenon, according to Heidegger, is “that which shows itself *as something* showing itself” [emphasis added] (Heidegger, GA 63, p. 67).^6^ In other words, and from an autonomy point of the view, even before one begins the process of reflecting on one’s values, desires, and motivations as part of one’s exercises of one’s autonomy, the phenomenological reduction implies that one has already recognised one’s values *as* values, *as* specific values, *as* one’s values, and *as* values that make sense in terms of the other attitudes and commitments that one holds as well as in terms of one’s lived experience. The point is that those aspects of oneself on which one draws to exercise one’s autonomy “are already operative in our everyday understanding” (Sheehan 2014, p. 128). Thus, if, as we shall argue in section two, those dimensions of the self that allow one to meaningfully access one’s characteristics and dispositions on the basis of which one can lead a self-determining and self-governing life are factors on which one’s capacity for, and exercise of, autonomy are dependent, then the phenomenological reduction requires us, as bioethicists, medical ethicists, health practitioners, and patients, to pay attention to, and account for, the conditions and structures of the self that allow one to encounter “being” in general, and the “being” of phenomena in particular, in terms of their meaningfulness in our everyday lives. This leads us to the point that, for the early Heidegger, meaningfulness is encountered through practical action, specifically, contextualized, first-person, embodied and affective experience (Sheehan 2014, pp. 128 − 30).

Like Heidegger, Merleau-Ponty rejected the idea of the phenomenological reduction as a reduction to “absolute consciousness” (Smith 2005). Indeed, many aspects of his approach to the phenomenological reduction echoed those of Heidegger: as he claimed, “Heidegger’s ‘*In‐der‐Welt‐Sein*’ [being-in-the-world] appears only against the background of the phenomenological reduction” (Merleau-Ponty 2002, pp. xiii–xiv). Firstly, our most immediate and fundamental mode of access to the world is not a cognitive act (ibid., p. x). Secondly, there is no sharp distinction between the inner and the outer, between self and world (ibid., p. 407). Thirdly, when it comes to the meaningfulness of our engagements in the world, intentionality initially manifests in active bodily engagement (ibid., pp. 138-9).

In terms of attempting to standardise an approach to the phenomenological reduction that considers one’s affective and embodied (i.e., lived) experiences, the approach taken here, which characterises our primary experiences as inherently and immediately meaningful, calls into question the traditional philosophical distinctions between rational and irrational, reason and emotion, and mind and body, which also have tended to characterise accounts of autonomy in medical decision making. By encountering meaningfulness, we do not impose a subjective interpretation on empirical data, nor do we passively sense or represent some sort of independent and isolable entity that determines meaning (see, for example, Heidegger, GA 2, pp. 190-1; GA 63, p. 3; GA 45, p. 85). Detached reflection of phenomena, including reflections on those aspects of the self that traditionally have been deemed to constitute the standards for autonomous decision making, presuppose inherently meaningful encounters with those same phenomena. Indeed, according to Heidegger, that meaningfulness can become “distorted” through rational reflection (Heidegger GA 17, p. 288). To even turn our own autonomy-constituting values, desires and motivations into objects of reflection is to *already* acknowledge the inherent meaningfulness of those attitudes and, consequently, the dependence of such reflective inquiry on our meaningful access to objects, which can be constituted as much by “irrational” feelings, emotions, moods, and bodily movements as by “rational” understanding (Heidegger, GA 2, p. 210). Central to phenomenological conceptions of autonomy, on which we are attempting to shed light, is the idea that, prior to taking up a detached reflective stance towards our own selves and each other, we are always already corporeally and affectively engaged in the world. Perception, bodily comportment, and reflection are (in varying degrees) embodied and skilful activities that allow us to cope with the world.^7^

## Pre-reflective Awareness and Bodily and Affective Intentionality

The conviction that is at work in our functional characterisation of the phenomenological reduction – one that is, in varying degrees, shared by classical phenomenologists – is that detached reflective self-awareness is only possible because there is a prior pre-reflective self-awareness built into experience. In autonomy terms, one’s motivating attitudes are always already meaningful even before one comes to reflect on or rationally respond to them. Furthermore, at least from Heidegger and Merleau-Ponty’s respective points of view, the upshot of the phenomenological reduction is that such meaningfulness is not immediately or fundamentally the result of a cognitive act, but, as intentional content (in the sense that it is “perpetually directed” at some “goal” or “project of the world” (Merleau-Ponty 2002, p. xx)), primarily manifests in bodily engagement and practical action.^8^ Indeed, for Heidegger, it also manifests through our affective experiences in the world. In this section, we extend the analyses of the phenomenological reduction in the works of Merleau-Ponty and Heidegger in order to explain the relationships between the capacities for bodily and affective intentional experience and the capacity for autonomy. In addition, we illustrate the ways in which manifestations of bodily and affective intentionality in terms of skilful, practical coping and moods, emotions, and feelings relate to exercises of autonomy. Finally, for both bodily intentionality and affective intentionality, we highlight some of the key implications for clinical decision making when it is recognised that autonomy is, in part, necessarily dependent upon the capacities for bodily and affective intentional experience and the exercise of those capacities.

### Bodily Intentionality and Autonomy

Pre-reflective self-awareness and the unity with the world that it encompasses is often to be understood in terms of one’s practical coping with the world (Crowell 2013, p. 174; Gallagher 2012, p. 78). An individual is intentionally involved with and in the world through active bodily engagements that cannot be equated with the deliberative outcome of her desires and beliefs. This approach to bodily intentionality, which Hubert Dreyfus finds in Merleau-Ponty and the early Heidegger, has been labelled as “absorbed coping,” that is, “the experience of a steady flow of skilful activity in response to one’s sense of the environment” whereby “one’s body is solicited by the situation to get into the right relation to it,” “something like what athletes call flow, or playing out of their heads” (Dreyfus 2014, p, 81; also see, Dreyfus 2000). As Wrathall (2015, p. 195) observes, “highly skilled, fluid actions are experienced…as being drawn out of me directly and spontaneously by the particular features of the situation, without the mediation of occurrent mental or psychological states or acts.” Challenging overly cognitive conceptions of autonomy, Meyers (2005, p. 40) makes a similar point; embodied engagement is a form of “practical intelligence,” yet one that “people seldom exercise self-consciously.” The point is that when we are “in the flow,” there is no cognitive disengagement between one’s behaviour and one’s surroundings; an individual is one with their setting and thereby aware of the skilfulness and unity of their body. In one sense, then, bodily intentionality can be taken to be the condition of “unity,” “equilibrium,” or “poise” in one’s environment (Kong 2017, pp. 78–81).

Kong (2017, pp. 51–99) has demonstrated the importance of the phenomenology of absorbed skilful coping for autonomy considerations in mental capacity assessments, particularly when such considerations involve cognitively-impaired patients who may be unable to satisfy the standard cognitive demands of capacity law. She argues that absorbed coping, like autonomy, is normatively significant in that “there is a sense of satisfaction when we manage to achieve it” (ibid., p. 80). Relatedly, in seeking to develop a phenomenology of illness, Fredrik Svenaeus argues that, on the basis of their illness, a patient’s body is experienced simultaneously as their own yet alien – obtrusively presenting itself to the patient as “broken” and “no longer under control” (2000b, p. 131). For Svenaeus, and based, in part, on what he considers to be an unorthodox interpretation of Heidegger’s concept of “*Unheimlichkeit*” as “unhomelikeness,” not only is illness an “uncanny” or “unhomelike” way of being-in-the-world (Svenaeus 2000a, b), but also the idea of “fixing” a patient means supporting her “back to a homelike being-in-her-own-body” (Svenaeus 2000b, p. 134). Echoing these points, Meyers (2005, p. 39) observes that “alienation from the body brought on by physical pain, illness, or injury” can be “profoundly disorienting.” Viewed in this light, not only do “ingrained bodily configurations and habitual bodily practices help to preserve one’s sense of self” (ibid.), but also, Meyers argues, authentic traits, affects, values, and desires (i.e., those attitudes that underlie one’s exercises of autonomy) can be “enacted” through absorbed coping in a way that “gives people the sense of wholeness that is characteristic of autonomy” (ibid., pp. 45 − 6). These comments raise the question of the nature of the relationship between, on the one hand, bodily intentionality, which is non-cognitive to the extent that it does not involve rational reflection on one’s values, desires, and motivations, and, on the other, those cognitive capacities and acts that have traditionally been the focus of philosophers and medical ethicists in accounts of autonomy. It is to this question that we now turn.

In light of a case study involving a severely autistic adolescent, not only does Kong observe how detached reflection can disrupt the flow of absorbed coping and the experienced equilibrium with the world, she illustrates how stereotyped or repetitive motor movements – acknowledged as “stimming” by autistic adults – serve as a crucial coping strategy for affective distress and the experience of bodily disunity (Kong 2017, pp. 51–99). Theoretical perspectives have suggested that individuals with autism find it difficult to govern their thoughts and actions because of excessive, insufficient, and inefficient sensory processing and perceptual inconstancy (Kapp, Steward, Crane et al., 2019, pp. 1782-3). Autistic adults have reported exhibiting repetitive, usually rhythmic bodily movements and vocalisations in response to distorted or overstimulating perceptions and dysregulated, excessive, or distracting thoughts, all of which are very often triggered by confusing, unpredictable, and overwhelming environments (ibid., p. 1786). They claim that stimming not only allows them to *cope with their environments*, but also affords them a level of control over their body, their affective states, and their thoughts (ibid., pp. 1788-9).

Psychological and theoretical perspectives on autism seem to validate the phenomenological principle that active bodily engagement, characterised above in terms of absorbed coping, is a necessary condition for meaningful pre-reflective experience. The point is that certain unpredictable or overstimulating environments disrupt the “equilibrium” or “poise” that individuals with autism would otherwise have in their environments, leading to experiences of bodily, affective, and reflective disunity (Kong 2017; Kapp, Steward, Crane et al., 2019). Viewed accordingly, stimming functions as a compensatory means of (re)establishing the “flow-like” experience of skilfully engaging with, and responding to, the environment, and, on that basis, affords a level of control over cognitive acts that, from an autonomy perspective, govern actions and choices (Kapp, Steward, Crane et al., 2019).

Although these discussions concerning the bodily intentionality of individuals with autism have specific implications for how phenomenological concepts could be employed in clinical decision-making contexts involving cognitively-impaired or neurodiverse patients, these implications lie beyond the scope of the current paper. Nevertheless, the principle that certain bodily movements – in this case, stimming – can re-establish a sense of bodily unity also extends to general clinical situations in which an ill patient should be supported by healthcare practitioners to experience “homelike being-in-her-own-body” such that her body “once again attains a central place in the patterns that make out her being-in-the-world” (Svenaeus 2000b, p. 134). As Meyers observes, one way in which experiences of bodily alienation resulting from pain, illness, or injury can be overcome is by, if possible, re-establishing “ingrained bodily configurations and habitual bodily practices” (Meyers 2005, p. 39), In terms of autonomy, then, a patient’s capacities for active bodily engagement or absorbed coping are not only, when interpreted in the context of the phenomenological reduction, necessary for pre-reflective self-awareness and inherently meaningful experience in general, but also necessary components of the capacity for autonomy (i.e., autonomous agency). In principle, individuals without those capacities would be unable to achieve the level of unity, equilibrium, or poise needed for reflective self-awareness.

As mentioned previously, there are principled reasons to distinguish between the capacity for autonomy and the exercise of autonomy. In terms of the latter, not only does the phenomenological reduction entail that bodily intentionality is necessary to meaningfully access the values, desires, and motivations to which one responds in exercising one’s autonomy, Meyers suggests that absorbed coping “enacts” these motivating attitudes in a way characteristic of the exercise of autonomy (Meyers 2005, pp. 45 − 6). For both Kong (2017, p. 80) and Meyers (2005, p. 46), bodily intentionality is normatively significant to the extent that it provides the conditions by which one can skilfully and intelligently navigate and respond to one’s environment in a way that is self-satisfying, or, according to Svenaeus (2000b, p. 134), by which one can experience “homelike being-in-one’s-own-body”. The fact that one finds lived bodily unity to be satisfying or “homelike” implies that the attainment of the state of “being-in-one’s-own-body” is something that one values, a value on which one will act at the pre-reflective level. Not only does this mean that bodily intentionality as it manifests in active bodily engagement is a necessary component of autonomous agency, but also it entails that the skills expressed through absorbed coping are, in part, constitutive of exercises of autonomy. Furthermore, these skills, as practical intelligence, not only respond to the value of lived bodily unity, they, as Meyers (2005, p. 48) argues, also can function to review, re-review, validate or disown other values, desires, and motivations. This non-cognitive dimension of the exercise of autonomy is often non-existent in abstract discussions regarding the conditions for autonomy typically found in traditional accounts. Nevertheless, in clinical reality, when practitioners are faced with patients who are experiencing a sense of bodily disunity as a result of pain, illness, or injury, the enactment of skills expressed through absorbed coping in a way that responds to a patient’s own values could be vital to the exercise of her autonomy. As Meyers claims, “we define ourselves as we act, and we cannot redefine ourselves without altering our patterns of action:” this “vocabulary is a trenchant vehicle for communicating avowal and disavowal and for advocating either persisting in or altering one’s course” (Meyers 2005, p. 46).

In terms of the implications of accepting bodily intentionality as a necessary component of the capacity for autonomy, and as a component, and constitutive of exercises of autonomy, firstly, the previous analysis suggests that clinicians should be attentive to the features of a patient’s search for equilibrium at the level of active bodily engagements in her environment. On the one hand, as Svenaeus (2000b, p. 134) argues, this means that treatments and healthcare support should strive to ensure that a patient can once again feel at home in her own body such that it “once again attains a central place in the patterns that make out her being-in-the-world” (even if such ways of being-in-the-world end up being drastically altered as a result of long-term or permanent changes to the patient’s lived bodily experiences as a result of her illness). On the other hand, if a patient is displaying bodily movements or vocalisations that a clinician might initially perceive to be heteronomous or irrational, then this could indicate that the patient is experiencing bodily or affective distress. Such distress could, in part, come down to the patient’s response to her illness, to proposed medical interventions, or to clinical decision making in general. It could also be the result of interactions between a patient and their clinician. And this idea finds conceptual support in Merleau-Ponty’s notion of *intercorporeity*, according to which the intentional contents of one’s bodily engagement “are not just formed in one’s individual body as the result of an isolated subjective process but depend in a dynamic way on the other’s elicitations and responses” (Gallagher 2012, pp. 199–200). Not only would it seem like good clinical practice for clinicians and healthcare staff to ensure that patients do not experience any undue stress caused by the clinical decision-making encounter, but also, on the basis that the model of Shared Decision Making (SDM) is now the *de facto* health policy standard for many national health providers (Lewis 2020), it is in the interests of both clinicians and their patients to ensure that the latter are supported by a decision-making environment that allows them to exert a level of control over their body and affective states in order to exercise their autonomy.

Secondly, if we accept Meyers’ argument that the skills displayed by individuals though absorbed coping can enact their values, desires, and motivations in a way characteristic of the exercise of autonomy (Meyers 2005, pp. 45 − 6), then there is an autonomy-based reason for practitioners to facilitate decision-making encounters that allow patients to exercise those skills and that provide the former with the opportunity to understand those skills and their relations to underlying values. This would involve operationalising the concepts of bodily intentionality and the conditions of absorbed coping for application in SDM contexts (for an example of how such concepts could be operationalised in the context of mental capacity assessments, see Kong 2017, pp. 51–99).

Thirdly, the phenomenology of absorbed coping provides a principled basis for calling into question attempts to eliminate motor stereotypies in both capacitous and incapacitated patients, attempts which remain popular both clinically and in research (Kapp, Steward, Crane et al., 2019). This coincides with the recent call for well-informed clinicians to pay greater attention to the “lived experiences” of patients living with long-term motor disabilities or somatic illnesses in order to facilitate interventions that allow them to lead fulfilling and autonomous lives *with* their symptoms (Humpston and Broome 2020) or that allow them to feel as “homelike” as possible in a new and different form of being-in-the-world than the one present before the onset of illness (Svenaeus 2000b, p. 135).

### Affective Intentionality and Autonomy

Turning now to the affective dimension of intentional experience and its relationship to autonomy, the point we should recall from earlier is that, according to typical theories of autonomy, whether and to what extent an individual can be said to “authentically” exercise their autonomy when making decisions and choices depends on whether and to what extent the values, desires, and motivations on which she reflects are her own. Similarly, at least according to more phenomenological readings,^9^ Heidegger, in working out his notion of authenticity, looks for those attitudes and experiences in which we discover ourselves to be an ineliminable ground of our decisions and actions. For Heidegger, the primary instances where we have the chance to see ourselves in this way are when we feel experience anxiety, when we are no longer gripped by social norms, and when we are conscious of our need to take responsibility (Braver 2014, p. 89; Crowell 2013, p. 204; Wrathall 2015, p. 206).^10^ From a phenomenological perspective, these experiences cognitively disengage us from our everyday fluid, skilful practices of absorbed coping, and entail that we must choose to make a choice and thereby take responsibility for our lives, which Heidegger calls “resoluteness” (Heidegger, GA 2, p. 297). Nevertheless, as we have already demonstrated, the reflective acts that these experiences entail still depend on bodily intentionality and the skills expressed through absorbed coping: “I articulate *courses of action* – weighing evidence and considering reasons for going on in one way or another” [emphasis added] (Crowell 2013, p. 202).

Transposing this idea into clinical decision-making contexts, when a patient suffers from a chronic illness, she may be, for pathophysiological reasons, unable to fluidly respond to the demands and solicitations of her environment in ways typical of her usual being-in-the-world. Accordingly, a patient may decide to choose the intervention that best restores the meaningful way she engaged with and in the world prior to becoming ill thereby maintaining her practical identity. For instance, if the patient is suffering from early-stage amyotrophic lateral sclerosis (“ALS”), she might choose a direct cognitive intervention, such as a brain-computer interface, on the basis that it can purportedly re-establish her capacities for absorbed coping and thereby maintain those skills expressive of her practical identity. Alternatively, a patient may decide to commit to a different way of being in the world and thereby choose the intervention that would best establish a new practical identity. For example, a patient who develops schizophrenia might choose to avoid clinical interventions that focus solely on correcting thoughts and perceptions in order to explore therapies that allow him to lead a fulfilling life with, yet in better control of, his symptoms.^11^ Whatever attitude the patient holds towards their practical identity, and, accordingly, whichever treatment option she chooses, the point is that when a patient meets with her physician in order to discuss treatment options, the cognitive disengagement from her active bodily engagements means that, at the cognitive level, she can choose to take those values that she initially disclosed at a pre-reflective level as “a reason for an action that necessarily figures in the explanation of that action as an action” (Wrathall 2015, 206).

On the face of it, Heidegger’s phenomenological approach to authentic, reflective self-awareness may appear to affirm more traditional conceptions of autonomy: it acknowledges that the capacity for reason is a necessary condition for the exercise of an individual’s authority over her actions; it recognises that an agent with the capacity for reason is able to effect changes in her life in a manner that is consistent with the sort of practical identity she would want to adopt; and it acknowledges that authentic choices are intentional and free from the direct grip of external influences. However, Heidegger is sceptical of our ability to cognitively control how we exercise our capacity for reason. As Braver (2014, p. 90) suggests, “even the limited form of autonomy that resoluteness can achieve is not something I can give myself.” For Heidegger, the point is that our power to exercise our autonomy is dependent on affective intentionality, that is, when it manifests in moods.

According to Heidegger, moods are “fleeting experiences that ‘colour’ one’s whole ‘psychical condition’” (Heidegger, GA 2, p. 450). Consequently, moods call into question the centrality of rational, introspective reflection in traditional approaches to autonomy. Rather than accept that reason belongs to thought alone, feelings and emotions are much more “rational” than we first thought (Heidegger, GA 5, pp. 5–10). This is because moods, emotions, and feelings, which Heidegger does not consistently distinguish between, not only determine how and in what way we react to the world and others, they are fundamentally entwined with the process by which we exercise our capacity for reason in authentic decision-making contexts. In short, moods are beyond the scope of rational reflection and self-governance because they are a condition of both. The upshot of Heidegger’s account of the conditions for authentic choice is that it reveals significant limits to our ability to cognitively determine how we exercise our autonomy. The phenomenological reduction and the phenomenological approach to moods “emphasises our passivity;” “we *find* ourselves in a mood not of our making or of our choice, and we can’t change our mood simply by deciding to do so” (Braver 2014, p. 53).

In clinical situations, a patient will not usually find herself affectively inert when she comes to make decisions and treatment choices. Under the influence of pain or drugs, or in response to a specific health condition or proposed medical intervention, she may temporarily experience shock, fear, panic, or fatigue during the clinical encounter.^12^ The point that we can take from Heidegger is that lived affective experience through moods, feelings, and emotions affect the way we make sense of our values, desires, and motivations when we reflect on or respond to them in exercises of our autonomy. “Good moods” may help us to perceive our attitudes as things that we really want to respond to and which we deem to be warranted and deserving of respect. Conversely, “bad moods” may lead us to feel alienated from our motivating attitudes or lead us to repudiate our values. As with bodily intentionality, not only does this mean that affective intentionality is a necessary component of the capacity for autonomy, but also it entails that moods, emotions, and feelings, which manifest affective intentionality, are, in part, constitutive of exercises of autonomy in the sense that they can function to approve or disown those motivating attitudes on which authentic choice is based.

Despite the fact our ability to control our moods and affective attitudes is limited, they can be affected indirectly by altering aspects of the meaningful contexts and relationships in which we find ourselves. This idea has received support in the relational autonomy literature.

Specifically, recognising the fact that malign interpersonal relationships can causally contribute to a patient’s inability to exhibit the necessary positive affective attitudes towards herself, Mackenzie (2008) argues that it is the physician’s obligation to empathise with the patient and support her to experience those affective attitudes that allow her to esteem, respect, or recognise her values as meaningful or valuable. The point that Svenaeus (2000b, p. 134) makes regarding health practitioners’ obligations to support the patient to return to a homelike being-in-the-world through the help of medicine also applies here. From a phenomenological perspective, achievement of the latter could be supported by facilitating a clinical decision-making environment that allows the patient to experience those moods and emotions through which she normally makes sense of the world in terms of her everyday lived experiences, and this may extend to affective attitudes of trust, esteem, and/or respect for herself, her motivating attitudes, and her decisions (Meynen 2011). In addition, if, as with bodily intentionality, a patient’s capacity for autonomy and the exercise of that autonomy are necessarily dependent on certain moods, emotions, and feelings, then it would seem like good clinical practice for clinicians and healthcare staff to ensure that patients do not experience any undue affective distress during the clinical decision-making encounter. Furthermore, given, as Svenaeus (2000b, pp. 134-5) suggests, that bringing a patient back to “homelikeness” such that she no longer experiences her own body in an obtrusively alien way may require her to adopt a new and different being-in-the-world and thereby a new and different practical identity, such an approach to clinical practice does not rule out the possibility of extending the decision-making process over several clinical encounters. As Martin and Hickerson (2013, p. 212) observe, this would ensure that due regard is given to a patient’s “temporal capacities” to “knit together [her] past experiences in such a way as to project [herself] meaningfully into an uncertain and existentially open future.” Furthermore, and although an affectively- and bodily-oriented conception of autonomy would, in principle, allow for authentic patient decisions that, from the outside, may appear to be irrational, heteronomous, or anomalous, a temporal approach that extends the decision-making process over several clinical encounters may, for clinicians concerned about whether a patient is genuinely exercising their autonomy, serve to illustrate that a patient’s judgments regarding their values, desires, and motivation are rational and authentic, and not subject to external or internal autonomy-undermining influences.

## Phenomenological Autonomy or Relational Autonomy?

It has been suggested that the phenomenological reduction validates the relational turn in the analysis of the concept of autonomy (Kong 2017, p. 81). We should recall that relational accounts of autonomy are premised on an understanding of interpersonal and social relationships as either constitutive of autonomous agency or the background conditions that causally affect the exercise and achievement of autonomy (Mackenzie 2021). In this final section, we consider the question of whether and the phenomenologically oriented approach to autonomy, which the phenomenological reduction entails, demands a commitment to relational autonomy.

In the previous discussions concerning Heidegger’s notion of authenticity, which grounds one’s decisions and choices in one’s being-in-the-world, we observed that, when it comes to medical decisions, a patient might choose a treatment option that best maintains the practical identity she had before she became ill. Alternatively, she might choose to pursue a new practical identity. Both approaches are compatible with Heidegger’s notion of authenticity. However, even if a patient wishes to re-establish or maintain her practical identity through medical treatment, there may be certain instances where, in the eyes of clinicians, such a practical identity appears to be heteronomous or irrational when considered in the light of current Western clinical and socio-cultural norms. For instance, commentators have highlighted the conflicts between the typical standards of modern, Western clinical practice and medicine and the “way of life” that grounds the practical identities of Ultra-Orthodox Jewish patients (Glick et al. 2011; Gabbay et al. 2017). Patient practices and behaviours based on Orthodox observance of “Halacha,” a practical code derived from the Hebrew Bible and the canon of rabbinic literature, can extend to avoidance of medical treatment or refraining from asking for healthcare assistance on the Sabbath, deference to rabbinic authority on the choice of intervention, family consultation in clinical decision making, and declining treatment or referral in cases of psychiatric illness (Gabbay et al. 2017). In addition, certain medical interventions, such as withholding of life-sustaining treatments in clinically futile cases, fertility therapy, invasive and non-invasive prenatal testing, abortions for foetal anomalies or maternal conditions, contraception, and the use of nonkosher products in medical care, may be deemed to conflict, or require reconciliation with the Halachic code (Glick et al. 2011). Even then, the delineation between Halachic and medical opinion is considered to be a complex and controversial topic (ibid.). For healthcare practitioners lacking in specific cultural competence or who do not seek to inquire about the unique religious, spiritual, and cultural factors that contribute to an Ultra-Orthodox Jewish patient’s practical identity, such approaches to medical treatments and clinical decision making may be perceived as irrational or symptoms of the patient’s condition, confused with a mental condition, or indicative of an absence of patient autonomy (Gabbay et al. 2017).

Relatedly, a clinician’s questioning of the rationality and the authenticity of a patient’s choice of treatment may also arise when, regardless of her specific religious, cultural, or social characteristics, she chooses to pursue a new practical identity. As Meyers (2005, p. 41) argues, a problem with applying principles of autonomy that appeal to rational reflection as its preeminent arbiter is that these principles provide scope to call into question the autonomy of “anomalous” decisions in the sense that they are perceived as departures from “certified patterns of behaviour:” “only after such anomalous behaviour had been scrutinized and judged to be expressive of authentic traits, affects, values, and desires could similar future behaviour count as autonomous.”

These issues emphasise the need for physicians and medical staff to be attentive to their patient’s search for equilibrium in her lived experiences, through which she displays skilful bodily engagements that constitute her practical identity, and which can enact those values, desires, and motivations that inform her choice of treatment. According to Kong (2017, p. 184; pp. 194-5), a physician’s attentiveness to the ways in which a patient’s illness has affected her practical identity and thereby her ability to fluidly cope with the world in the way she would want will help to establish a “shared perceptual understanding” on the basis of which a patient’s choices and associated reasons can be identified. Without these bonds of commonality, there is no possibility for seemingly “discordant” behaviour or “anomalous” treatment choices to be recognised as anything other than irrational or heteronomous. This can be damaging, particularly for patients with motor disabilities or somatic illnesses, or for patients who adopt practical identities in accordance with their specific cultural or religious values that seem incompatible with the general norms governing modern clinical practice and medical treatment. It can not only lead to clinical encounters that generate conflict and undue bodily, affective or reflective distress and disunity (see, for example, Gabbay et al. 2017), and pave the way for therapeutic interventions that seek to correct seemingly irrational behaviour thereby “entrench[ing] oppressive, disabling practices” (Kong 2017, p. 185), but also disregard, constrain, or manipulate a patient’s practical identity and thereby her ways of engaging and coping with the world (ibid., p. 197). In addition, it implies a failure on the part of the physician to recognise the patient as someone who has the *status* of autonomy. This can disable the patient’s ability to exercise her autonomy and, simultaneously, facilitates the conditions for unwarranted paternalistic intervention (Lewis 2021b).

What these discussions demonstrate is that a phenomenological approach to the *exercise of autonomy* (i.e., one that takes into account the relationships between affective and bodily intentionality and authentic choice) is compatible with one particular approach to relational autonomy, that is, one that includes *causal* relational conditions for autonomy (Holroyd 2009). The point is that although one may fulfil the affective, bodily, and cognitive conditions for autonomous agency, how one exercises one’s autonomy when making choices and decisions will, in part, depend on interpersonal relationships, including whether a clinician takes into account a patient’s practical identity as it relates to her autonomy, whether a clinician respects the exercises of a patient’s autonomy, and whether a clinician recognises the patient as someone who has the status of being an autonomous agent. Furthermore, to the extent that a patient’s capacities for bodily and affective engagement are, when interpreted in the context of the phenomenological reduction, necessary for reflective engagement with one’s values and thereby necessary for the capacity for autonomy (i.e., autonomous agency), by facilitating clinical decision-making environments that generate undue bodily, affective, or cognitive distress, clinicians can contribute to a patient’s inability to fulfil the conditions for the capacity for autonomy. What makes such accounts of autonomy *causally* relational is that, as Christman (2004) demonstrates, interpersonal relationships contribute to an agent’s psychological states (and, as we have sought to demonstrate in this article, their affective states and embodied experience) and are thereby part of the “background requirements” for autonomy. In other words, certain interpersonal relations might *cause* an agent to fulfil or fail to meet the cognitive and non-cognitive conditions for autonomous agency or choice (Holroyd 2009).

Relational accounts of autonomy do not only consist of the causal variety (Mackenzie 2021). An account is considered to be “exclusively,” “uniquely,” or “thoroughly” relational if it posits particular normative social and interpersonal relationships as “conceptually necessary requirements of autonomy” (Christman 2004, p. 147). A thoroughly relational view of autonomy presumes the metaphysical claim that autonomy is a property of the relations that comprise social conditions in which an agent is embedded (ibid., p, 159). If that is the case, then being autonomous means not only satisfying the first-person capacities for autonomy, but also being embedded in the right kinds of social conditions with the right kinds of interpersonal relationships. Rather than causally relational, such accounts are taken to be “constitutively” relational (Holroyd 2009).

There is nothing in or entailed by the phenomenological reduction to suggest that certain social and interpersonal conditions are necessary, as constitutive conditions, for a phenomenologically oriented conception of autonomous agency. As we have seen, the phenomenological reduction implies that one has, prior to reflective engagement, already pre-reflectively experienced one’s values *as* values, *as* specific values, and *as* one’s values through the manifestation of one’s bodily and affective intentionality. In other words, the phenomenological reduction is the basis of experience and, accordingly, the experience of autonomy. Neither making sense of one’s experience by employing the phenomenological reduction nor the exercises of those bodily and affective capacities that provide pre-reflective, meaningful access to aspects of oneself to which one responds when making decisions and choices require one to stand in any particular social or interpersonal relationship. Furthermore, it is implausible that the phenomenological reduction necessitates constitutive social conditions for one to either experience cognitive disengagement from one’s everyday absorbed coping through which one discovers oneself to be the ground of one’s decisions and actions, or to decide upon a treatment choice in response to those grounds disclosed at a pre-reflective level. As we have seen, a patient can be seen to make “anomalous” decisions and exhibit “discordant” or “inconsistent” behaviour, or pre-reflectively experience their values, desires, and motivations through embodied engagements and affective states that appear heteronomous or irrational. On the basis of the phenomenological reduction, these are all compatible with phenomenological conceptions of the conditions for autonomy and authentic choice.

## Conclusion

The phenomenological reduction implies that moods and embodied practical coping are vital to the disclosure of the meanings of things, people, and events. In autonomy terms, this means that even before one begins the process of reflecting on one’s values, desires, and motivations as part of one’s exercises of one’s autonomy, one has pre-reflectively experienced one’s values *as* values, *as* specific values, *as* one’s values, and *as* values that make sense in terms of one’s practical identity. On that basis, and by extending the analyses of the phenomenological reduction in the works of Heidegger and Merleau-Ponty, this paper has argued that the affective and embodied dimensions of experience are, in principle, integral parts of a patient’s capacity for, and exercise of autonomy, and should, therefore, be accorded appropriate recognition by healthcare providers and practitioners in decision-making contexts. These arguments reveal that traditional individualistic theories of autonomy, relational theories, and standard accounts of autonomy in biomedical ethics and medical law, to the extent that they assume that autonomy is purely conditioned by cognitive capacities and processes of introspective self-reflection, are, in practice, ill-suited to fully capture patient autonomy, particularly when patients’ experiences of pain, illness, or injury result in experiences of bodily or affective distress or disunity. Finally, whereas some have suggested that the phenomenological reduction validates a relational conception of autonomy, we argued that a phenomenological approach to autonomy, grounded as it is by the phenomenological reduction, is not compatible with relational autonomy when the latter assumes that interpersonal and social relationships are constitutive conditions of autonomy.

## Footnotes

1 According to Tom Beauchamp and James Childress (2013, pp. 104-5), a patient’s choices are rendered autonomous when they are made intentionally, free from controlling influences and with adequate understanding of medically-relevant information. Adequate understanding is also a core component of the General Medical Council’s ethical guidance. 

2 There, are, of course, exceptions in the relational literature. According to “strong substantivists” (see, for example, Oshana 1998), an individual’s endorsement of, identification with, or rational response to attitudes, beliefs, and reasons is largely irrelevant if she does not commit to an externally defined, non-authoritarian value structure. However, the majority of relational theorists do not share the conviction that social conditions are constitutively necessary conditions of personal autonomy.

3 To this end, we rely on the approaches adopted in the Anglo-American tradition of phenomenological exegesis by Lee Braver, Steven Crowell, Thomas Sheehan and Mark Wrathall.

4 On the basis that Husserl’s notion of the phenomenological reduction seems to require a retraction from the world, it has often been held that Heidegger and Merleau-Ponty’s respective commitments to the notion of “being-in-the-world” are incompatible with the phenomenological reduction.

5 As Smith (2005, n. 3) observes, although Merleau-Ponty’s conception of “*être au monde*” should not be conflated with Heidegger’s “*In‐der‐Welt‐Sein*”, there are obvious affinities between the two. Here, we employ a notion of “being-in-the-world” based on those affinities, rather than account for the specific interpretive nuances of and between the two.

6 This idea is also found in Husserl’s work, who claims that consciousness is consciousness of something *as something* in the sense that our engagements with phenomena deliver an “interpretive sense” (Husserl 2001, 121).

7 This is in contrast with, for example, the interpretation of perception as an intermediary in a two-step, realist epistemological model, whereby passive receptions of something like sense data are synthesised as representations of external objects.

8 See, for example, Merleau-Ponty (2002, p. 162): “Our bodily experience of movement is not a particular case of knowledge; it provides us with a way of access to the world and the object, with a ‘*praktognosia*’, which has to be recognised as original and perhaps as primary.”

9 Here, we draw attention to the distinction between more phenomenological readings of Heidegger, as provided by Lee Braver, Steven Crowell, Thomas Sheehan and Mark Wrathall, and “layer-cake” pragmatist readings adopted by, for example, Hubert Dreyfus, Mark Okrent, and John Haugeland. According to “layer-cake” interpretations, our meaningful, norm-governed, practical coping is both pre-predicative and nonconceptual and, therefore, distinct from assertoric and propositional intentionality.

10 For a discussion of how this sort of disengagement from one’s everyday being-in-the-world that is necessary for authentic understanding relates to the “uncanny” or “unhomelike” disengagement from one’s lived body, which one experiences through illness, see Svenaeus 2000b, pp. 127–131.

11 See, for example, Humpston and Broome 2020.

12 At common law, it has been held that such “temporary factors” may lead to a temporary erosion of a patient’s capacity for autonomy (see, for example, *Re MB (Medical Treatment)* ([1997] 2 FLR 426, at [30]).

## Statements and Declarations

No ethical approval necessary.
